# Upregulated Wnt-11 and miR-21 Expression Trigger Epithelial Mesenchymal Transition in Aggressive Prostate Cancer Cells

**DOI:** 10.3390/biology9030052

**Published:** 2020-03-09

**Authors:** Elif Damla Arisan, Ozge Rencuzogullari, Ines Lua Freitas, Syanas Radzali, Buse Keskin, Archana Kothari, Antony Warford, Pinar Uysal-Onganer

**Affiliations:** 1 Institute of Biotechnology, Gebze Technical University, Gebze 41400, Kocaeli, Turkey; damlaarisan@gmail.com; 2 Department of Molecular Biology and Genetics, Istanbul Kultur University, Atakoy Campus 34156, Istanbul, Turkey; ozgeberrak@gmail.com (O.R.); keskinbuse19@gmail.com (B.K.); 3 Cancer Research Group, School of Life Sciences, College of Liberal Arts and Sciences, University of Westminster, 115 New Cavendish Street, London W1W 6UW, UK; w1667723@my.westminster.ac.uk (I.L.F.); w1667005@my.westminster.ac.uk; (S.R.); tony.warford@gmail.com (A.W.); 4 Department of Histopathology, Kingston Hospital, Galsworthy Road, London KT2 7QE, UK; archana.kothari2@nhs.net

**Keywords:** microRNA, in situ hybridization, prostate cancer, Wnt-11, miR-21

## Abstract

Prostate cancer (PCa) is the second-leading cause of cancer-related death among men. microRNAs have been identified as having potential roles in tumorigenesis. An oncomir, miR-21, is commonly highly upregulated in many cancers, including PCa, and showed correlation with the Wnt-signaling axis to increase invasion. Wnt-11 is a developmentally regulated gene and has been found to be upregulated in PCa, but its mechanism is unknown. The present study aimed to investigate the roles of miR-21 and Wnt-11 in PCa in vivo and in vitro. First, different Gleason score PCa tissue samples were used; both miR-21 and Wnt-11 expressions correlate with high Gleason scores in PCa patient tissues. This data then was confirmed with formalin-fixed paraffin cell blocks using PCa cell lines LNCaP and PC3. Cell survival and colony formation studies proved that miR-21 involves in cells’ behaviors, as well as the epithelial-mesenchymal transition. Consistent with the previous data, silencing miR-21 led to significant inhibition of cellular invasiveness. Overall, these results suggest that miR-21 plays a significant role related to Wnt-11 in the pathophysiology of PCa.

## 1. Introduction 

Prostate cancer (PCa) is the development of carcinoma within the prostate gland of the male reproductive system. It is a heterogeneous disease and may present itself as an indolent or aggressive form [1]. In most cases of PCa, the disease progresses at a slow rate, and symptoms often only occur at later stages when it is metastasized to secondary sites within the body, such as the bones, often leading to mortality. Overall, 10-year survival rates within patients are at an average of 84%, but this decreases with age (Cancer Research, UK). Currently, there is no specific form of screening for PCa, and health practitioners may use a combination of several tests in order to diagnose a patient. A grading system known as the Gleason score (GS) is in use as a guide, alongside several other factors, in order to determine the aggressiveness of cancer and the potential type of treatments that may be required [2]. Tumors with lower GS indicate cells that are similar to normal cells and are likely to be less aggressive. In contrast, those assigned with higher scores are indicative of tumors with poorly differentiated cells, and they are more aggressive [3]. The prostate-specific antigen testing (PSA) is another method used in screening for PCa; however, this method has raised some controversy, as it has been previously associated with over-diagnosis and false-positive results in patients, because it is not PCa-specific. Therefore, more specific biomarkers are required for PCa diagnosis and prognosis. 

MicroRNAs (miRs) are highly conserved small non-coding RNAs that control gene expression and regulate biological processes by targeting messenger RNAs (mRNAs). miRs can inhibit or enhance mRNA degradation [4]. Oncogenic miRNAs promote tumor development and often lead to poor prognosis [5]. miR-21 is one of the oncogenic miRs located on chromosome 17q23.1 and often found to have an altered expression profile in many cancers, including PCa [6]. miR-21 is thought to play an important role in the survival of the tumor cell via metastasis and proliferation, such as regulating tumor-suppressor gene phosphatase tensin homolog (PTEN) in PCa [7,8]. The phosphatase product of PTEN acts to dephosphorylate the phosphatidylinositol 3,4,5 triphosphate (PIP3), which, in turn, leads to the inhibition of the PI3K/AKT signaling pathway, preventing uncontrolled cell proliferation [9]. Various studies have shown the transfection of miR-21 into metastatic PCa cell line PC3 resulted in a further increase in cell viability and proliferation, as well as invasion [10]. In addition to this, decreased expression of PTEN in cells transfected with miR-21 confirms miR-21 negatively regulates PTEN. Moreover, functional studies of miR-21 revealed its function as an antiapoptotic factor across various cell types, whereby the expression of miR-21 was found to directly influence the upregulation of antiapoptotic genes such as Bcl-2 and Survivin, alongside pro-proliferation genes known as MMP2 and MMP9 [11].

Wnt-11 is a member of the Wnt signaling pathway, which is activated during embryogenesis for normal development, and recently, its upregulation was found in various malignancies, including PCa [12,13,14]. Wnt-11 is found to be upregulated in metastatic disease, and more importantly, it has been shown that it involves in TGF-β signaling pathways during the epithelial-to-mesenchymal transition (EMT), which is important for prostate cancer cell migration and invasion [13]. Several miRNAs have been reported as being associated with Wnt-signaling pathway genes [15]. A recent study by using meta-analysis showed that miR-21 is a good diagnostic biomarker for PCa [16]; both Wnt-11 and miR-21 were linked to EMT [17]. 

Currently, androgen (AR) deprivation therapy remains as one of the main therapeutic approaches for PCa [15]. However, this method of therapy is found to have a short period of positive response within patients, as they eventually progress into a castration-resistant state [18]. At this stage, cancer becomes AR-insensitive and is no longer responsive to deprivation therapy. miR-21 has been found to be particularly upregulated in castration-resistant PCa [19]. Furthermore, elevated miR-21 in LNCaP cell lines proved to be sufficient in rescuing cells from AR-ablated growth arrest and instead allowed AR independent growth [20]. Moreover, miR-21′s contribution to AR receptor-negative PCa cells showed that, by partially inhibiting the expression of miR-21, the growth of AR-independent PC3 and DU145 cells became impaired [20]. Taken together, the results from these studies reaffirm the role that miR-21 may play in PCa and its potential not only as a biomarker but also as a therapeutic target. In this study, we aimed to elucidate if Wnt-11 and miR-21 are associated at the first in a clinical setting in vivo and in vitro.

## 2. Materials and Methods

### 2.1. Cell Culture 

PCa cell lines LNCaP and PC3 (ATCC, Manassas, VA, USA) were maintained in Roswell Park Memorial Institute (RPMI) 1640 (Gibco-Life Technologies, CA, USA) with 10% (v/v) heat-inactivated fetal bovine serum (Pan Biotech, Aiedenbach, Germany) and penicillin-streptomycin (10,000 units penicillin/mL and 10 mg streptomycin/mL) (Pan Biotech, Germany) at 37 °C in a humidified 5% CO_2_ incubator (Hera Cell 150i, Thermo, USA).

### 2.2. In Situ Hybridization, Cell Blocks, and Immunostaining

PCa sections were purchased from UCL Biobank and provided as formalin-fixed, paraffin-embedded, and sectioned for histological analysis. Representative hematoxylin-eosin-stained sections were examined to evaluate the histopathological characteristics of the lesion to be analyzed. All samples were independently reanalyzed for Gleason grade by a pathologist (A.K). Sections were deparaffinized and rehydrated using graded alcohol concentrations. Tissue permeabilization was performed using protease-K 2–15 μg/mL (Sigma-Aldrich, UK). Slides were washed in diethylpyrocarbonate (DEPC) water and incubated in prehybridization solution for 60 min at 37 °C, then an equal volume of miR probe (dig-labeled miR-21/scrambled—250 ng/μL), and incubated overnight. After washing with Tris-buffered saline (TBS), slides were then incubated with anti-digoxigenin alkaline phosphatase antibody (1:600) (Roche, Burgess Hill, UK) for 60 min at room temperature. Slides were washed with TBS and then alkaline phosphatase buffer, followed by the addition of the NBT/BCIP substrate solution (Sigma Aldrich, Dorset, UK) with 1 μL of 1 μM levamisole, and slides were incubated overnight. Finally, the slides were washed in water and mounted with aqueous mountant. Cells harvested from PCa cell lines LNCaP and PC3 then were collected in phosphate-buffered saline (PBS) solution. Each specimen was centrifuged for 5 min at 2700 g twice, and the pellet was suspended with 100 µL PBS. Gelatin solution (10%) was prepared, and 0.5 mL was added to the cell pellets. After hardening, each cell block was transferred into glass tubes containing neutral buffered formalin (NFB). Following washing, the blocks were kept in 70% alcohol in 4 degrees. Then, the blocks were immersed with 100% alcohol for 60 min and xylene for 45 min twice before embedded in paraffin at 60 °C for 45 min. Finally, the cellblocks of the cell lines were cut at 5 μm and used in the in-situ hybridization method to detect miR-21, as described above. For Wnt-11-staining, antigen retrieval was performed by incubation in 10 mM sodium citrate pH 6.0 and heating in a microwave oven at 560W for 8 min. Endogenous peroxidase was quenched using 3% H_2_O_2_ for 30 min. Following blocking (30 min in PBS with 1.5% horse serum), sections were incubated with goat anti-Wnt-11 at 1:200 for 1 h at room temperature. Washing and antibody visualization was done using the Vectastain Elite ABC Standard kit according to the manufacturer’s instructions (Vector Labs, Southgate, UK). Tissue samples were scored by A.W. for miR-21 and Wnt-11 levels in PCa epithelial cells (0 = no signal, 1 = positive, and 2 = strongly positive).

### 2.3. RNA Extraction and qRT-PCR 

RNA was extracted from cells using Trizol (Sigma, Haverhill, UK), and RNA concentration and purity were measured using the NanoDrop spectrophotometer (Thermo Fisher Scientific, Hemel Hempstead, UK) at 260 nm and 280 nm absorbance. RNA was reverse-transcribed to cDNA using the qScript microRNA cDNA Synthesis Kit (Quantabio, Lutterworth, UK) according to the manufacturer’s instructions. The resulting cDNA was used to assess the expression of miR-21, while RNU6 and hsa-let-7a-5p were used as a reference RNA for normalization of miRNA expression levels, as described before [21]. Each experiment was repeated six times in duplicates. The relative expression of miR-21 was normalized with RNU6 and let-7 expressions using the comparative cycle threshold method [22]. In addition, the standard deviation was calculated as well as a *t*-test using Graphpad Prism 7.00 (La Jolla, CA, USA) software.

### 2.4. Assays for Colony Formation and Soft Agar Colony Formation Spheroids 

Anti-miR-21 (60 nM) transfection was performed on the LNCaP and PC3 cell lines. Briefly, anti-miR-21 transiently transfected cells were seeded at 1 × 10^4^ density in 6-well plates and incubated for 14 days. After media was removed and cells were washed with 1x PBS solution, they were fixed with methanol:acetic acid (3:1) for 20 min at room temperature. Then, the removal of fixing agents: cells were stained with 0.5% crystal violet in methanol for 15 min and were washed by distilled water, and the morphological images were taken under light microscopy. For the soft agar colony formation assay, 0.5% agarose and 2× RPMI at a ratio of 1:1 were mixed and applied to the bottom layer of the 6-well plates. Once the lower layer of agar had solidified, the upper layer was prepared. Then, in the microfuge tube was prepared a total volume of 1 mL (0.3% agarose solution, 5 × 10^4^ cells in 2× medium), and the solution was placed on the solidified agar. The plate was incubated at 37 °C in an incubator for 21 days. Colony formation potentials were examined by light microscopy. The diameter of the spheroid formation was analyzed by using the Olympus Micro DP Manager Image Analysis program in a time-dependent manner and figured out by graphics using a 4.04 version of GraphPad software.

### 2.5. Assays for Cell Invasion, Migration, and Apoptosis 

Cell invasion assays were performed as described before [20]. PC3 cells were transfected with anti-miR-21 after 48 h, and 5 × 10^5^ transfected cells were plated on Matrigel-coated Transwell filters (BD Biosciences, Nottingham, UK) in growth medium containing 1% FCS. After a further 24 h, the number of invaded cells was determined by crystal violet assay. Wound-healing cell migration assay was performed briefly by seeding the cells in a monolayer, then scratched with the narrow end of a sterile 10 µL pipette tip. The width of the scratch was measured at different points in each well after initial wounding. The cells were incubated for 24 h at 37 °C in a CO_2_ incubator, and then the scratch width was measured. The relative motility and migration ability of the cells into the cell-free zone is expressed as the normalized percent change in the scratch width after 0, 24, and 48 h.

### 2.6. Hanging Drop Assay 

PC3 cells were counted and calculated to be 25 × 10^2^ each drop (for the Anti-miR-21 were used anti-miR-21 transfected cells). The cells were seeded in a 60-mm plate lid as 10 µL drops. Plate lid was gently flipped upside down and place on a plate filled with 3 mL 1× PBS (to humidify the drops). The cells were incubated at 37 °C in a CO_2_ incubator. Three-dimensional spheroid formations were examined under a light microscope for 96 h with every 24 h calculation for randomly selected at least 10 spheroids. After 72 h, each drop was stained with DAPI and DiOC6 and examined under fluorescence microscopy (Olympus, Tokyo, Japan).

### 2.7. Boyden Chamber Assay

Cell migration was assayed by using modified Boyden chambers with an 8-μm pore Track-etched polyethylene terephthalate (PET) membrane (Fisher Scientific, Loughborough, UK). Cells/well (25 × 10^4^) were seeded after transfection protocols were proceeded in a 200-µL serum-free medium in the chamber. Medium (750 µL) (+serum) was added to the lower chamber. Cells were incubated at 37 °C in CO_2_ for 12–24 h. Cells were fixed with 3.7% formaldehyde for 2 min at room temperature (RT). Formaldehyde was removed, and cells were washed with 1x PBS. Methanol (100%) was used for permeabilization for 20 min at RT. Cells were washed with 1× PBS twice and stained with crystal violet. Stained cells were visualized by light microscopy at ×100 magnification. Cells were counted for five random fields and analyzed. 

### 2.8. Western Blotting

miR-21 transfected-LNCaP and PC3 cells were scraped with 1× PBS. Then, the cells were washed with ice-cold 1× PBS and lysed in M-PER mammalian protein extraction reagent (Thermo Scientific, Waltham, MA, USA) with protease inhibitor cocktail. After lysis, cells were centrifuged for 20 min at 16,000g at 4 °C, and total protein concentrations were determined with a Bradford protein assay (Bio-Rad, Hercules, CA, USA). Protein isolation procedure also proceeded for DU145 and PC3 spheroids as 3D cell cultures. Then, 30-µg of total protein was separated on 12% sodium dodecyl sulfate-polyacrylamide gels (SDS-PAGE) and transferred difluoride (PVDF) membranes (Thermo Scientific, Whaltham, MA, USA). Following the washing of membranes in Tris-buffered saline with Tween-20 (TBS-T) (10 mM Tris-HCl, pH.8.0, 0.05% Tween-20), PVDF membranes were blocked by 5% skim milk containing TBS-T milk 1 h at room temperature. Then, PVDF membranes were incubated in primary antibody buffer containing 5% (v/v) skim milk solution (E-cadherin (1:1000), β-catenin (1:1000), LRP6 (1:1000), Wnt-11 (1:100), Dvl-2 (1:1000), Axin-1 (1:1000), and β-actin (1:1000) (Cell-Signaling Technology, Danvers, MA, USA) overnight at 4 °C. Then, membranes were rinsed with TBS-T and incubated with horseradish peroxidase (HRP)-conjugated secondary antibodies (anti-rabbit IgG or anti-mouse IgG (Cell-Signaling Technology, Danvers, MA, USA) for overnight at 4 °C. Membranes were developed with an enhanced chemiluminescence reagent. All proteins were quantified relative to the loading control β-actin.

### 2.9. Data Analysis

All data were analyzed as means ± standard errors. Statistical significance was determined using a Student’s *t*-test or ANOVA with a Newman-Keuls post-hoc analysis, as appropriate. Results were considered significant for *p* < 0.05. Two-way ANOVA Bonferroni’s multiple comparisons test was performed using GraphPad Prism version 7.00 for Windows (GraphPad Software, La Jolla, CA, USA) www.graphpad.com. Western blotting results were analyzed with Image J, and obtained results were statistically analyzed with GraphPad Prism version 7.00 (La Jolla, CA, USA).

## 3. Results

In this study, four sets of results are presented. First, we describe the miR-21 and Wnt-11 expression profiles of different Gleason score patient samples; we demonstrate that both miR-21 and Wnt-11 expressions correlate with high Gleason scores in PCa patient tissues. Second, we show that miR-21 affects cell survival and colony formation. Third, we demonstrate that mir-21 orchestrates the epithelial-mesenchymal transition. Fourth, we reveal that silencing miR-21 expression results in significant inhibition of cellular invasiveness. Overall, these results suggest that miR-21 plays a significant role in the pathophysiology of PCa.

### 3.1. miR-21 and Wnt-11 Expression Profiling

**Figure 1:**
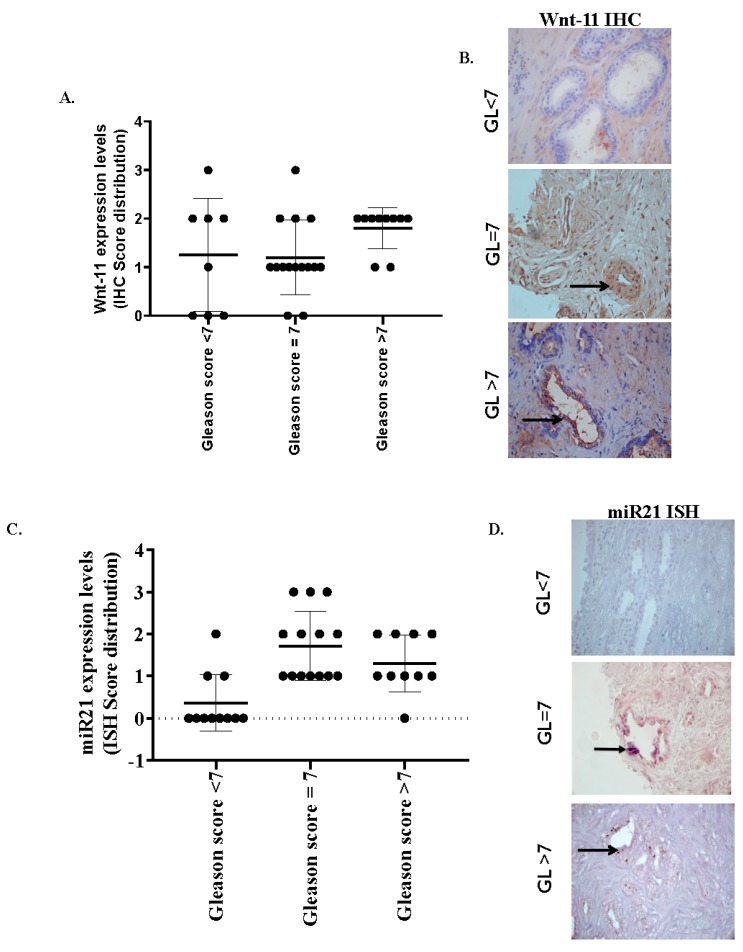
Wnt-11 protein expression in prostate cancer (PCa). (**A**) Gleason score distribution of Wnt-11 expression shows Wnt-11 expression significantly correlates with Gleason grade (*p* < 0.05 between three groups) (**B**) Immunohistochemical analysis of Wnt-11: (i) low Gleason score, faint cytoplasmic expression (black arrow) and stromal smooth muscle (red arrow) are indicated; (ii) an example of Gleason grade 4 tumor; and (iii) a strongly positive Gleason 5 tumor showed upregulated cytoplasmic expression (scale bar 100 μm). (**C**) miR-21 expression distribution increases with the Gleason score (*p* < 0.05 between three groups) (**D**) In-situ hybridization of miR-21-staining in a low Gleason score: (i) no miR-21-staining in tumor cells (black arrow), (ii) upregulated miR-21 expression on an example of a Gleason grade 4 tumor, and (iii) a strong expression of an miR-21 Gleason 5 tumor (scale bar 75 μm). IHC: immunohistochemistry and ISH: in-situ hybridization.

Wnt-11 protein levels are elevated in patient tumors; we used anti-Wnt-11 antibodies to localize Wnt-11 expression in sections taken from prostate tumor tissue samples. Twenty Gleason scores of 7 in PCa tissue samples were used for this study, as well as ten Gleason scores higher than 7 and ten Gleason scores lower than 7 in tissue samples. Immunohistochemical analysis of Wnt-11 in tumor tissues indicated that the level of Wnt-11 was elevated in 25/36 (69.4%; Figure 1A,B). The overall scores were presented in Table 1. The level of Wnt-11 expression was generally higher in Gleason scores of 7 in PCa samples compared to lower Gleason scores in prostate samples (Figure 1A). Analysis in relation to conventional prognostic indices of PCa showed a negative correlation with the Gleason grade; Wnt-11 was more frequently found in Gleason 7 grade 4 tumors, suggesting that Wnt-11 perhaps is an important marker during the mid-stage of cancer. 

**Figure 2:**
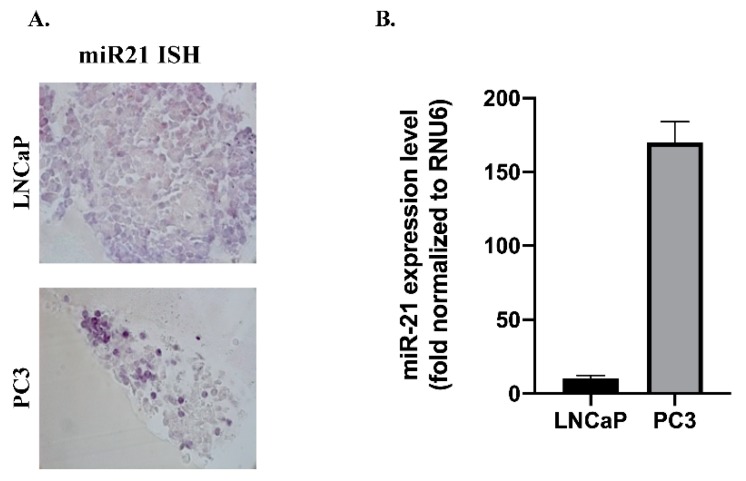
In-situ hybridization of miR-21-staining in PCa formalin-fixed paraffin cell blocks. (**A**) LNCaP (upper panel) cell blocks show less miR-21 expression and PC3 cell blocks (lower panel) indicate upregulated miR-21 expression correlates with metastatic behavior (*n* = 6). miR-21-staining was scored according to the staining intensity by a histopathologist (A.W.). (**B**) qRT-PCR analysis of expression levels of miR-21 in LNCaP and PC3 PCa cells (*n* = 3; *p* < 0.01). Data was normalized according to RNU6 and hsa-let-7a-5p.

Similarly, miR-21 was variably expressed in the tumor epithelial cells (Figure 1C,D and Table 1). Nonspecific staining was observed when a scrambled probe was used in all samples. Formalin-fixed paraffin cell blocks were prepared using LNCaP and PC3 PCa cell lines. miR-21-staining of PC3, a strongly metastatic PCa cell line, was intense compared with the weakly metastatic LNCaP cell line (*n* = 6; Figure 2A). The same cell lines were used to analyze miR-21 expression by qRT-PCR; upregulated miR-21 expression was noted in PC3 (120-fold compared to LNCaP cells; *n* = 6; *p* < 0.01) cells, thus correlating with ISH results (Figure 2B). Taking the data together, we conclude that further investigation of miR-21, segregated by the Gleason stage, may prove useful in assessing patient prognosis. 

### 3.2. miR-21 Involves in Cell Survival and Colony Formation

**Figure 3:**
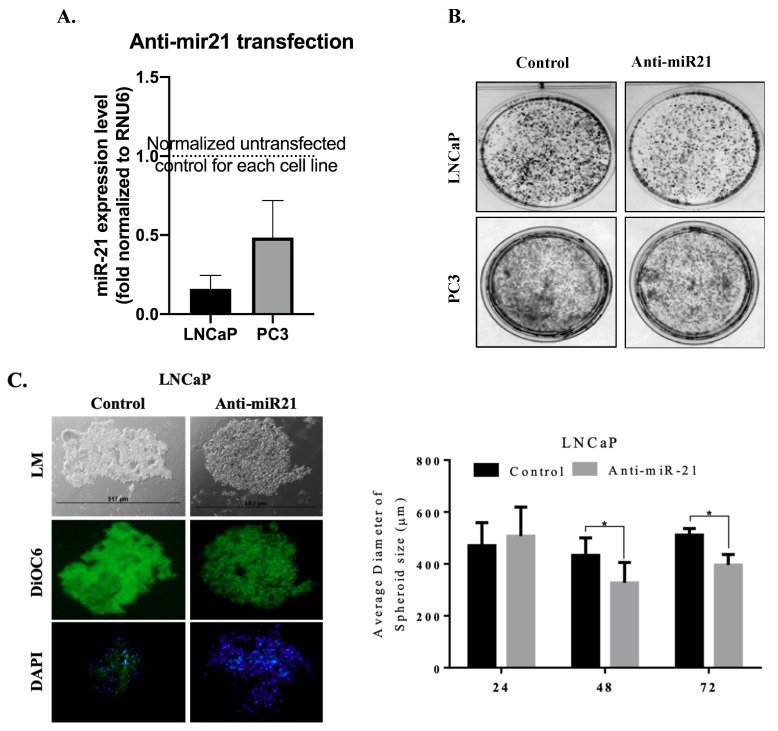
Downregulation miR-21 increased cell death in 2D and 3D cell culture environments. (**A**) Anti-miR-21 transfection reduced the expression levels of miR-21 in LNCaP and PC3 cells. Obtained data was normalized with RNU6 levels in each cell lines. (**B**) The long-term effects of miR-21 inhibition on cell proliferation and colonization was investigated by colony formation assay in LNCaP and PC3 cells. (**C**) Hanging drop assay was performed in order to investigate the effects of the inhibition of miR-21 in spheroid cultures. miR-21 downregulation increased apoptotic cell populations in 3D spheroids of LNCaP cells. Spheroids were stained by DiOC6 (viable cells) and DAPI (nuclei). Scale bar 100 µm. Columns represented the average ± std. dev. of with at least 3 separate experiments with ten repeats.

**Figure 4:**
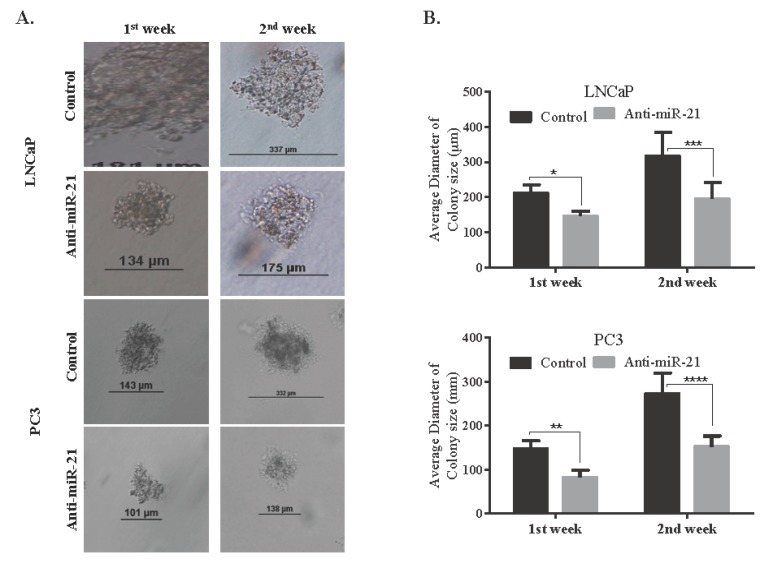
Long-term miR-21 downregulation decreased spheroid formation in LNCaP and PC3 PCa cells. (**A**) Soft agar assay was performed to evaluate the long-term effects of anti-miR-21 on LNCaP and PC3 cells for 7 and 14 days. (**B**) The reduction of colony formation size was analyzed with Tukey’s multiple comparison test (* *p* = 0.0367, ** *p* = 0.0013, and *** *p* = 0.0002). Columns represented the average ± std. dev. of with at least 3 separate experiments with 5 separate colonies.

**Figure 5:**
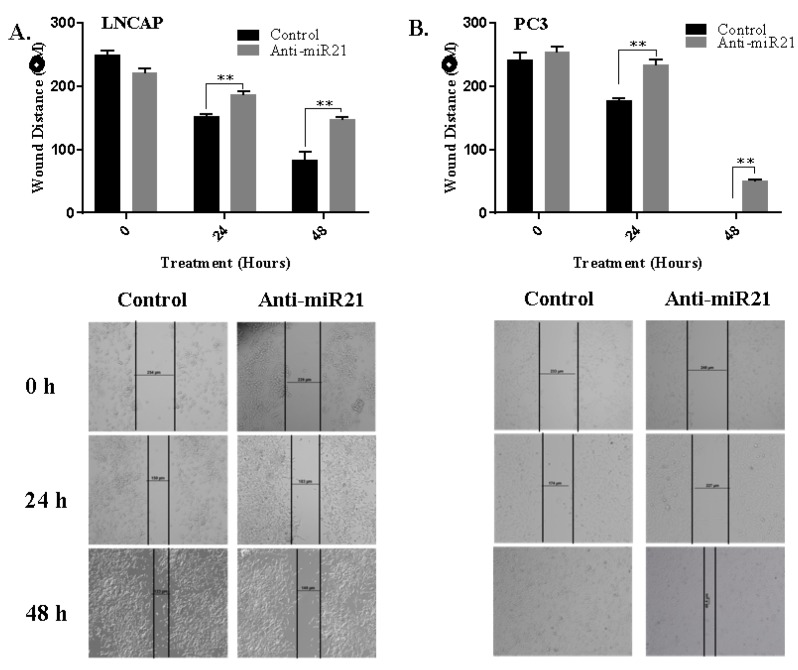
miR-21 downregulation reduced the wound-healing capacity of LNCaP cells more effectively than PC3 cells. Wound-healing assay was performed to determine the effects of anti-miR-21 on the migration capacity of (**A**) LNCaP and (**B**) PC3 cells. Wound closures of cells were photographed and analyzed periodically for 0–48 h. Columns represented the average ± std. dev. of at least 3 separate experiments (LNCaP, ** *p* = 0.0029 and PC3, ** *p* = 0.0036).

In order to evaluate the potential role of miR-21 on cell survival and on the ability to form colonies, we transfected cells with anti-miR-21. The efficiency of transfection was determined by analyzing the intracellular level of miR-21 expression. For the normalization of miR-21 expression levels, RNU6 C*_t_* values of each cell line were used as internal controls. The expression levels of miR-21 were more significantly reduced in LNCaP cells than PC3 cells. Following, we determined colony formation potential within 14 days to evaluate the miR-21-related cell proliferation mechanism. The inhibition of miR-21 resulted with a significant decrease on colony formation potential in LNCaP cells when compared with PC3 cells (Figure 3B). Additionally, the effect of miR-21 on tumor growth potential was investigated in 3D spheroid cultures. The hanging drop assay was performed to obtain spheroids in LNCaP cells (Figure 3C). The images shown in Figure 3C described the spheroids that were observed 72 h after seeding. Viable cells were determined by fluorescence microscopy after staining with DiOC6. Diminished miR-21 expression resulted in a significant increase in the apoptotic population within the core region of the spheroids, which were observed with light microscopy (Figure 3C). DAPI-staining consisted with a higher number of cells with DNA damage in the core region. In a similar way, soft agar assay was performed to observe the effect of miR-21 inhibition on the 3D colony cultures in LNCaP and PC3 cells. The inhibition of miR-21 significantly prevented colony formation in a cytostatic manner in both LNCaP and PC3 cells within 14 days, while untreated cells were growing (* *p* = 0.0367, ** *p* = 0.0013, and *** *p* = 0.0002) (Figure 4A,B). To be able to understand the antiproliferative and antimigratory effects of anti-miR-21 in LNCaP and PC3 cells, we utilized a scratch assay to understand the proliferation capacity of cells in the wounding site (Figure 5A,B). While anti-miR-21 treatment in LNCaP cells was effective to reduce wound closure in a time-dependent manner, PC3 cells were more aggressive against anti-miR-21 treatment within 48 h.

### 3.3. miR-21 is a Strong Regulator on EMT Pathway

**Figure 6:**
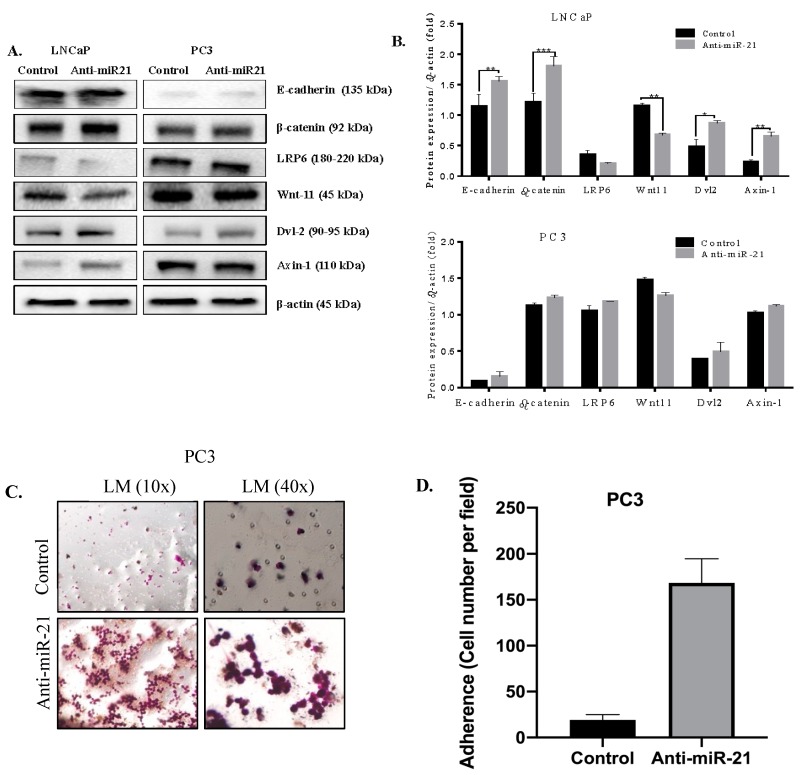
miR-21 modulated EMT and Wnt-signaling-related molecular targets in LNCaP and PC3 cells. (**A**) Total protein was isolated following transfection of anti-miR-21 for 48 h in LNCaP and PC3 cells. Western blotting was performed in order to determine expression levels of EMT-related molecular targets in LNCaP and PC3 cells. (**B**) Expression levels were analyzed by Bonferroni’s multiple comparison test; * *p* < 0.05, ** *p* = 0.0013, and *** *p* = 0.00018. β-actin was used as a loading control. (**C**,**D**) Boyden chamber assay was performed with anti-miR-21-transfected PC3 cells. Unmigrated cells were visualized by crystal violet staining, and cells were counted per fields that were randomly selected at ×100 magnification.

To clarify the molecular mechanism of anti-miR-21 transfection, we determined the E-cadherin expression profile of LNCaP and PC3 cells. E-cadherin expression levels of LNCaP were higher than PC3 cells, and anti-miR-21 transfection increased E-cadherin expression levels in LNCaP cells but not in PC3. β-catenin expression levels upregulated only in LNCaP cells; also, there was a slight increase in PC3 cells following anti-miR-21 transfection. Untreated PC3 cells showed higher expression profiles of Wnt-signaling axis-regulator protein LRP than LNCaP cells. While LRP6 was downregulated in anti-miR-21-transfected LNCaP cells, miR-21 inhibition upregulated LRP6 slightly in PC3 cells. Wnt-11 expression was lower in LNCaP than PC3 cells, since miR-21 inhibition caused a decrease in Wnt-11 expression. Although the downregulation of Wnt-11 was examined in miR-21-inhibited PC3 cells, it was not significant, as in LNCaP cells. Another Wnt regulator Dvl-2 upregulated following miR-21 inhibition in each cell line. Axin levels increased with miR-21 inhibition in each cell line but further in LNCaP cells (Figure 6A,B). Thus, we concluded that miR-21 expression levels are crucial in the decision of EMT in PCa prognosis. To confirm the anti-EMT effect of anti-miR-21 treatments, we utilized the Boyden chamber experiment, which is used for the invasion capacity of cells, as shown in Figure 6C. While the majority of cells were motile and alive according to DiOC6-staining results in PC3 control samples, anti-miR-21 transfection prevented the invasion of cells from micro pores on Boyden chambers.

## 4. Discussion

The main results were as follows: (1) Wnt-11 and miR-21 expression occurred commonly in PCa tissues, and they were significantly higher in Gleason score 7 compared with lower Gleason score tissues. (2) miR-21 promoted cell survival and colony formation. (3) miR-21 orchestrated EMT, as well as cellular invasiveness, without affecting proliferative activity. Recent studies highlighted that embryonic signals, which are activated during carcinogenesis, renders therapy potential and increased metastasis risk, which leads to increased mortality rates due to cancer progression. According to the protein atlas database, Wnt-11 is one of the embryonic signals activated during carcinogenesis that leads to poor progression [14]. We have previously reported that Wnt-11 leads to neuroendocrine differentiation of different PCa cell lines. Moreover, Wnt-11 orchestrates a number of proteins, including PKC, JNK, NF-κB, Rho, PKA, and PI3K [23,24]. Next-generation sequencing data-mining results from metastatic sites of castrate-resistant PCa patients show that there are mutations of Wnt signaling pathways in 18% of cases [25].

In this study, we found that Wnt-11 and miR-21 expression correlated with Gleason grade. Many studies demonstrated that miRNAs regulate the Wnt signaling pathway during embryogenesis, osteoblast differentiation, and bone formation, as well as cardiac development [26,27,28]. PCa has a high tendency to metastasize to bone, and currently, bone metastasis remains incurable, and therapies are limited [29]. It has been reported that FZD8 was robustly upregulated in bone-metastatic PCa cell lines and tissues, and FZD8 upregulation was significantly positively associated with clinical tumor progression and bone metastasis. FZD8 is a major Wnt-11 receptor, and Wnt-11 involvement was previously reported in PCa migration and invasion. Moreover, it was demonstrated that silencing FZD8 reduced EMT [30]. Crosstalk between TGFβ and Wnt signals has been studied extensively [31]. Furthermore, TGFβ expression is often increased in cancer cells, where it promotes EMT, metastasis, and chemoresistance. Similarly, miR-21 has been found to be upregulated in many carcinomas, including PCa and TGFβ/BMP4-induced pri-miR-21-processing, and upregulation of the mature miR-21 was also observed in MDA-MB-468 breast carcinoma cells [32]. Another study demonstrated that overexpressing FZD8 promotes, whereas silencing FZD8 suppresses, PCa cell migration, invasion, and stem cell-like phenotypes in vitro through an association of FZD8 and the TGFβ receptor complex [29]. Our results are in line with the findings that miR-21 may regulate Wnt-11 expression, perhaps via the FZD8-TGFβ complex. Previous studies also demonstrated that PTEN is one of the major gene targets of miR-21. PC3 cells do not express PTEN; however, LNCaP do. PTEN plays a significant role during cell survival and migration signaling and inhibits PI3K/AKT, as well as the focal adhesion-related signaling axis [33,34,35]. We have found that, after miR-21 was inhibited, the colony formation of LNCaP cells were decreased, but there was no clear effect on PC3 cell colony formation. Surprisingly, miR-21 inhibition alone did not affect cell proliferation of PCa cells. This is in line with other published studies that the downregulation of miR-21 had no additive effect on the inhibition of cell proliferation of doxorubicin-treated A549 and OVCAR3 cells, whereas there was an increase in sensitivity in SNB19 glioma cells [36]. In another study, overexpression of miR-21 caused resistance to gemcitabine of SUIT-2 and Panc-1 pancreatic cancer cells due to increased activity of PI3K/AKT/mTOR signaling as a consequence of the high suppression of PTEN by miR-21 [35]. Therefore, the inhibitive effect of anti-miR-21 on cell survival might be cell type-dependent. In our study, we have found that the inhibition of miR-21 more efficiently suppressed the invasion potential of LNCaP cells compared to PC3 cells. It is known that LNCaP cells are derived from lymph node metastasis, and PC3 cells are derived from androgen-independent bone metastasis. Additionally, PC3 cells have a higher metastatic potential than LNCaP cells [37]. Jin et al. suggested that bone morphogenetic protein receptor II (BMPRII) is one of the targets of miR-21 and also showed that the protein expression level of BMPRII conversely regulated with the level of miR-21 in LNCaP and PC3 cells. BMPRII played an important role during stem cell differentiation and cell proliferation. Therefore, miR-21 has potential in the regulation of the metastatic and malignancy abilities of PCa cells by BMPRII [38]. We have observed a remarkable effect of miR-21 inhibition on the anchorage-independent colony growth in LNCaP cells, which is consistent with previous results. Although hanging drops did not affect miR-21 inhibition, colony diameters were significantly reduced in PC3 cells. The inhibiting colony formation in each cell line suggested that miR-21 maintained the stem cell function and migration capacity of PCa cells. In non-small cell lung cancer, (NSCLC)-derived cells showed elevated levels of miR-21 in anchorage-independent growth conditions [39]. Therefore, miR-21 upregulation might explain one of the triggering mechanisms in malignant tumors. Our wound-healing results were also consistent with previous findings in LNCaP and PC3 cells, which were accompanied by the upregulation of epithelial marker E-cadherin expression levels in miR-21-inhibited LNCaP and PC3 cells [35]. However, β-catenin levels did not affect the inhibition of miR-21 in LNCaP cells, but a marked decrease in β-catenin levels was observed in PC3 cells. Moreover, increased expression levels of miR-21 further induced the migration of glioma cells, which are associated with the upregulation of Sox-2 and β-catenin protein [40]. Therefore, that might be the reason for the resumption of migration in LNCaP cells. While LRP levels were diminished by the inhibition of miR-21 in LNCaP cells, there was the opposite effect in PC3 cells. The downregulation of LRP was important during the prevention of migration, because LRP played an important role with CD44 in adhesion mechanisms in tumor progression [41]. Previous studies determined that the c-Jun transcription factor was associated with pre-miR-21 promoter regions [42]. Prevention of the activity of JNK-1 as a main activator of p-Jun decreased miR-21 expression and increased the other important target gene, PDCD4 (tumor-suppressor gene programmed cell death 4). In ovarian cancer cells, targeting the miR-21 resulted in the downregulation of cell proliferation through the diminishing of the JNK-1 pathway. In addition, blocking miR-21 overwhelmed the cisplatin resistance in ovarian cancer cells [43].

We have utilized the TCGA dataset (PRAD), which includes 568 PCa patients. Following the selection of the prostate cancer dataset from TCGA, we ran UCSC Xena to evaluate the relationship between Wnt11 and the Gleason score. Wnt11 was critical in the disease progression. Kaplan Meier datasets showed that an increase in the copy number of Wnt11 caused a lesser progression-free survival and decreased the overall survival rate. In addition, we have found that higher Gleason score patients have the highest expression levels of Wnt11 (prostate cancer Gleason Wnt-11 file). We confirmed these findings by a MEXPRESS view of the TCGA dataset, which indicates that Wnt11 is a critical target in prostate cancer disease progression (Supplementary File from TCGA).

## 5. Conclusions

Taken together, in this study, we showed that miR-21 plays an important role during cell migration and invasion. The EMT pathway was downregulated by the inhibition of miR-21, which demonstrated that miR-21 was a probable target to inhibit cell invasion but in a cell type-dependent manner. So, an efficient modulation of the downstream targets in the therapeutic scenario of miR-21 might depend on distinct regulators such as PTEN, β-catenin, or other cell migration-associated genes. Overall, these findings indicated that Wnt-11 and EMT pathways are maintained by miR-21. Further studies are needed to identify related signaling axes; however, our data and others indicate that miR-21 could be a useful target for PCa.

## Figures and Tables

**Table 1:** Summary of Wnt-11 and miR-21-staining results according to Gleason scores.

Histological Grade	Wnt-11-Staining 25/36 (69.4%)	miR-21-Staining 25/36 (69%)
Gleason < 7 (*n* = 10)	1/10 (10%) strong/moderate 1/10 (10%) weak 8/10 (80%) cases nonspecific	1/9 (10%) strong/moderate 2/10 (20%) weak 7/10 (70%) negative/nonspecific
Gleason 7 (*n* = 20)	8/17 (48%) strong/moderate	7/17 (42%)
	7/17 (42%) weak	7/17 (42%) weak
	2/17 (10%) nonspecific	3/17 (16%) nonspecific
Gleason > 7 (*n* = 10)	3/9 (33%) strong/moderate	4/9 (44%) strong/moderate
	5/9 (56%) weak	4/9 (44%) weak
	1/9 (11%) nonspecific	1/9 (11%) nonspecific

## Supplementary Materials

The following are available online at https://www.mdpi.com/2079-7737/9/3/52/s1.
