# Supplementary material for: Upregulated Wnt-11 and miR-21 Expression Trigger Epithelial Mesenchymal Transition in Aggressive Prostate Cancer Cells
Source: Biology (Basel). 2020 Mar 9;9(3):52. doi: 10.3390/biology9030052 (PMC7150874; doi:10.3390/biology9030052)
Supplement: Supplementary file 1 [file biology-09-00052-s001.pptx]

## Slide 1
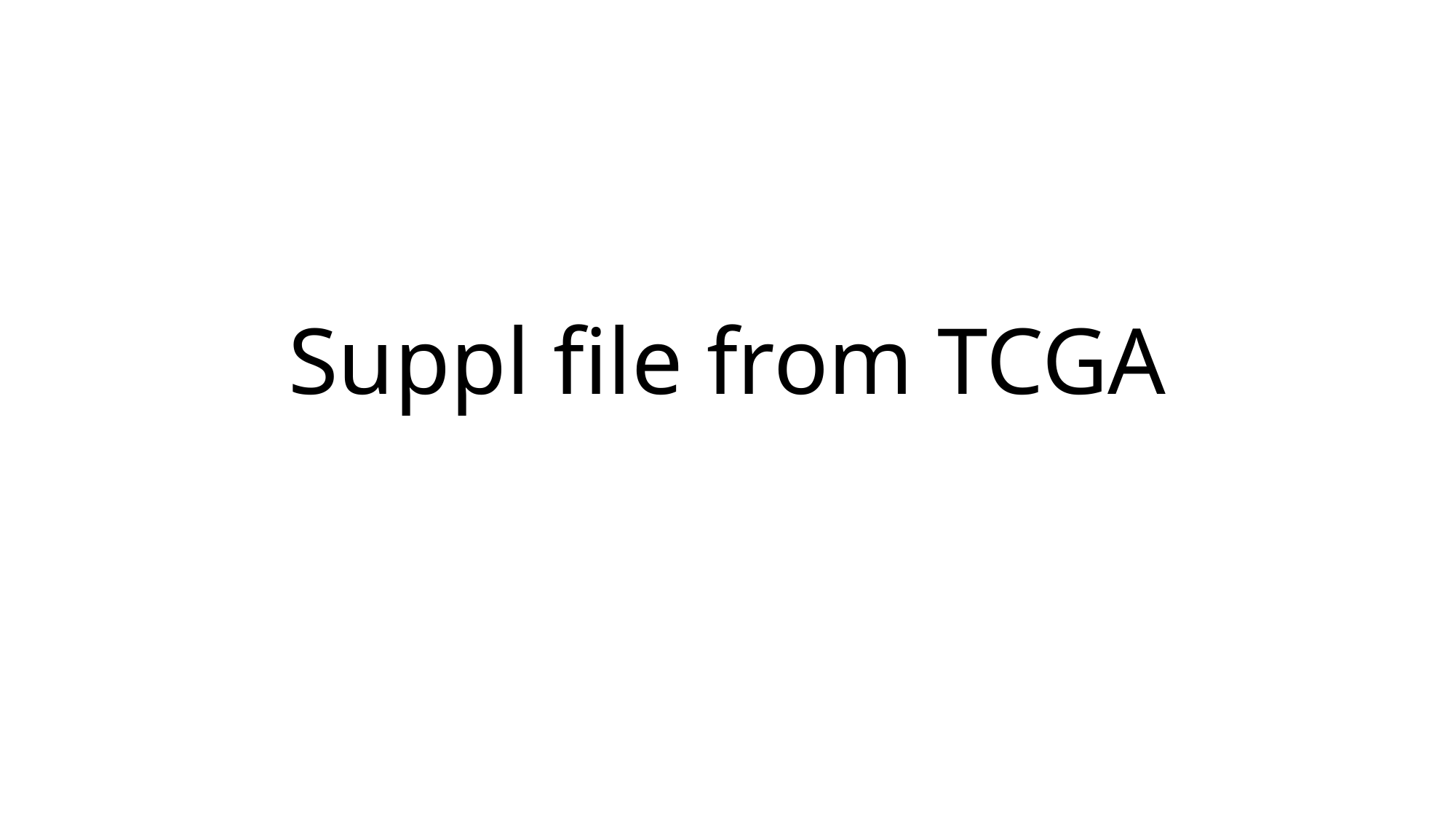

# Suppl file from TCGA

## Slide 2
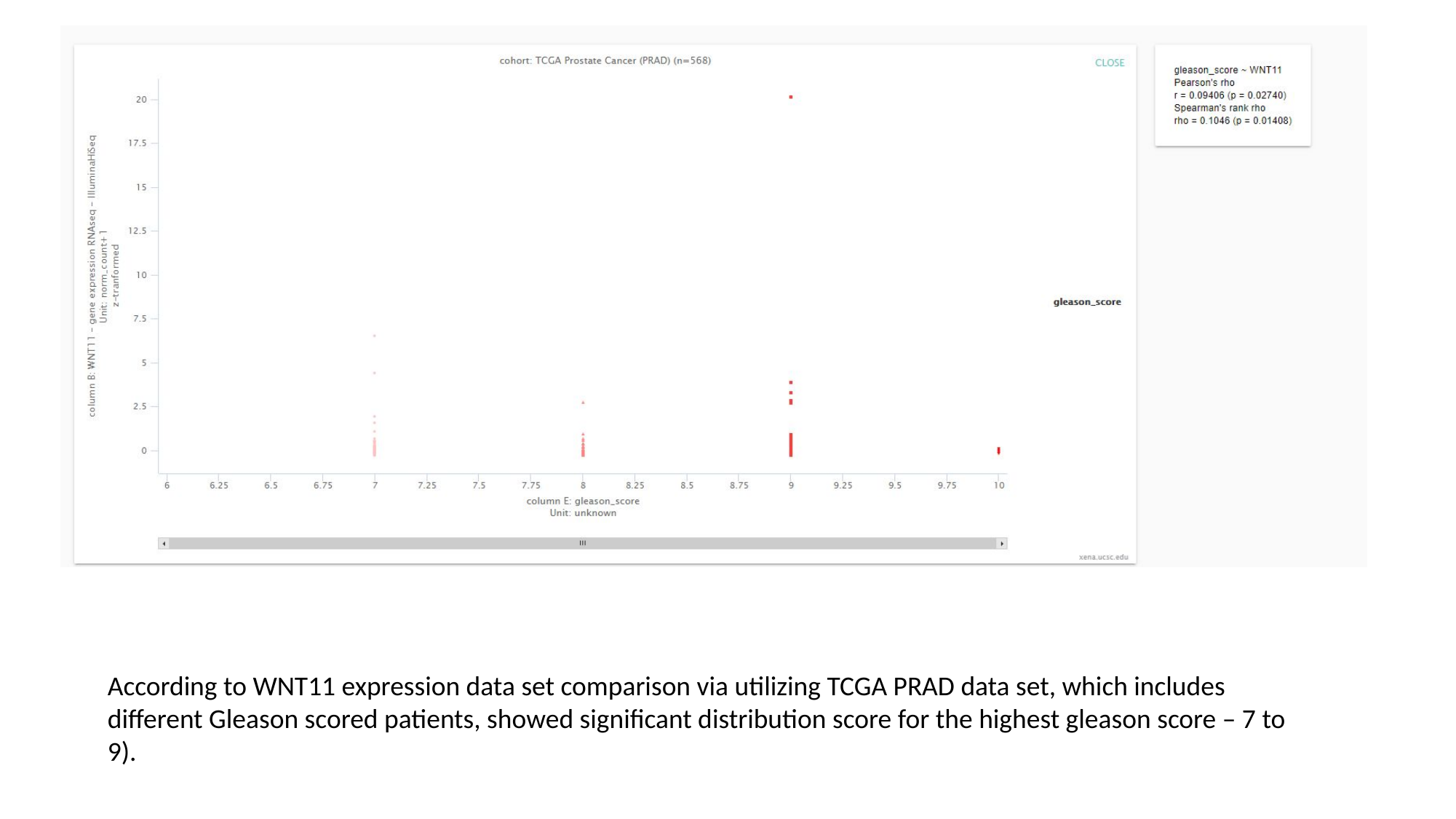

According to WNT11 expression data set comparison via utilizing TCGA PRAD data set, which includes different Gleason scored patients, showed significant distribution score for the highest gleason score – 7 to 9).

## Slide 3
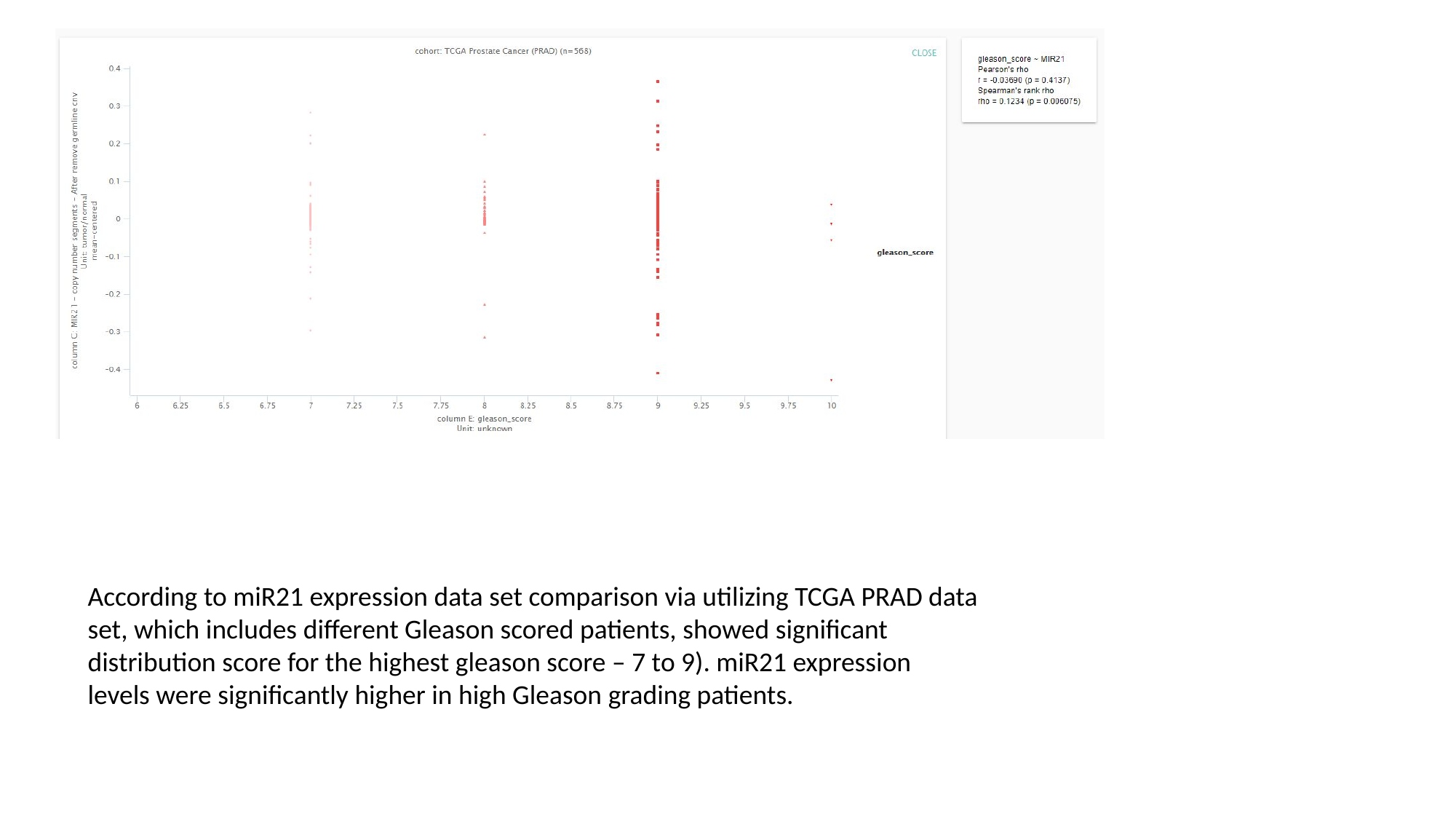

According to miR21 expression data set comparison via utilizing TCGA PRAD data set, which includes different Gleason scored patients, showed significant distribution score for the highest gleason score – 7 to 9). miR21 expression levels were significantly higher in high Gleason grading patients.

## Slide 4
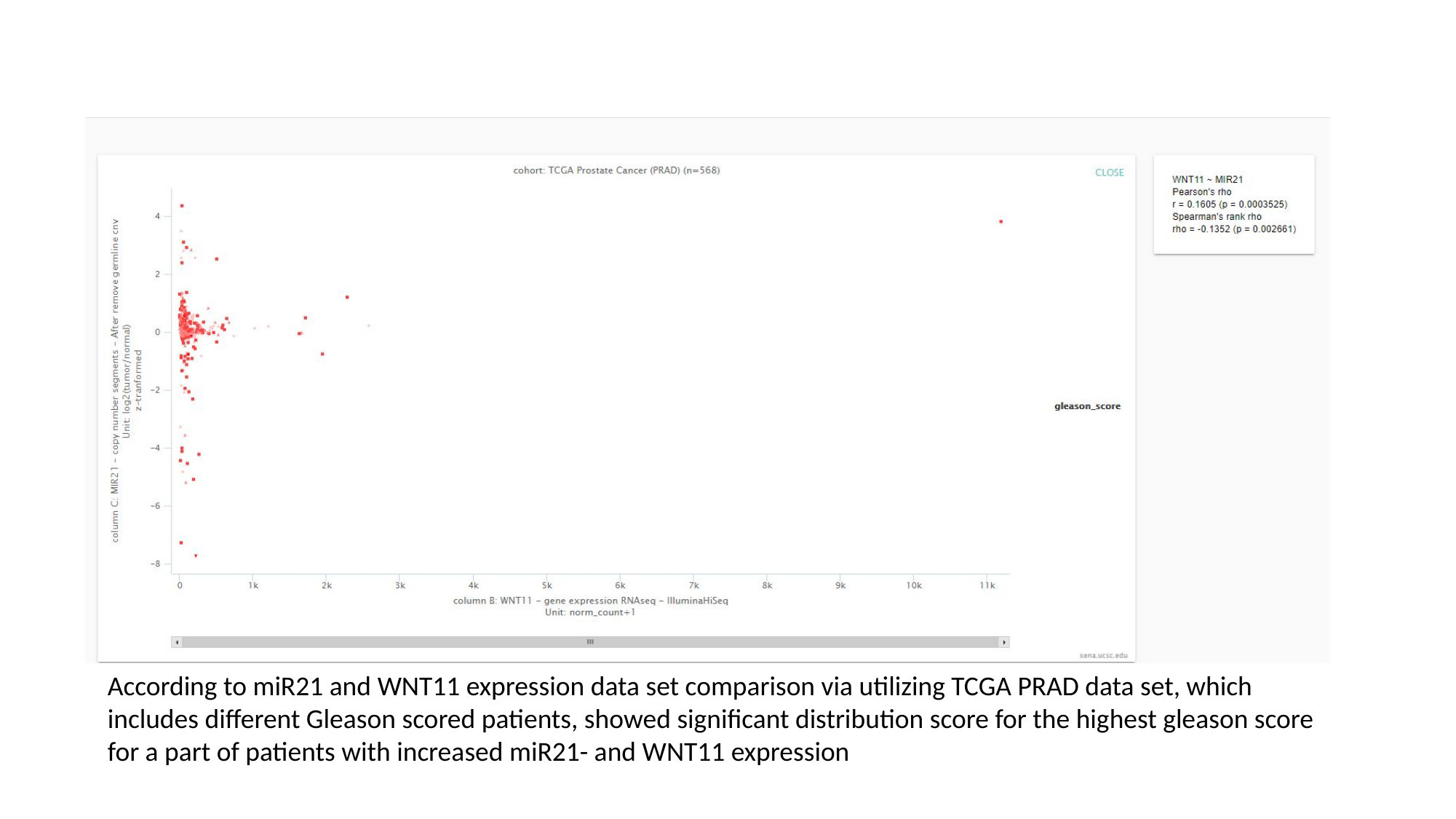

According to miR21 and WNT11 expression data set comparison via utilizing TCGA PRAD data set, which includes different Gleason scored patients, showed significant distribution score for the highest gleason score for a part of patients with increased miR21- and WNT11 expression

## Slide 5
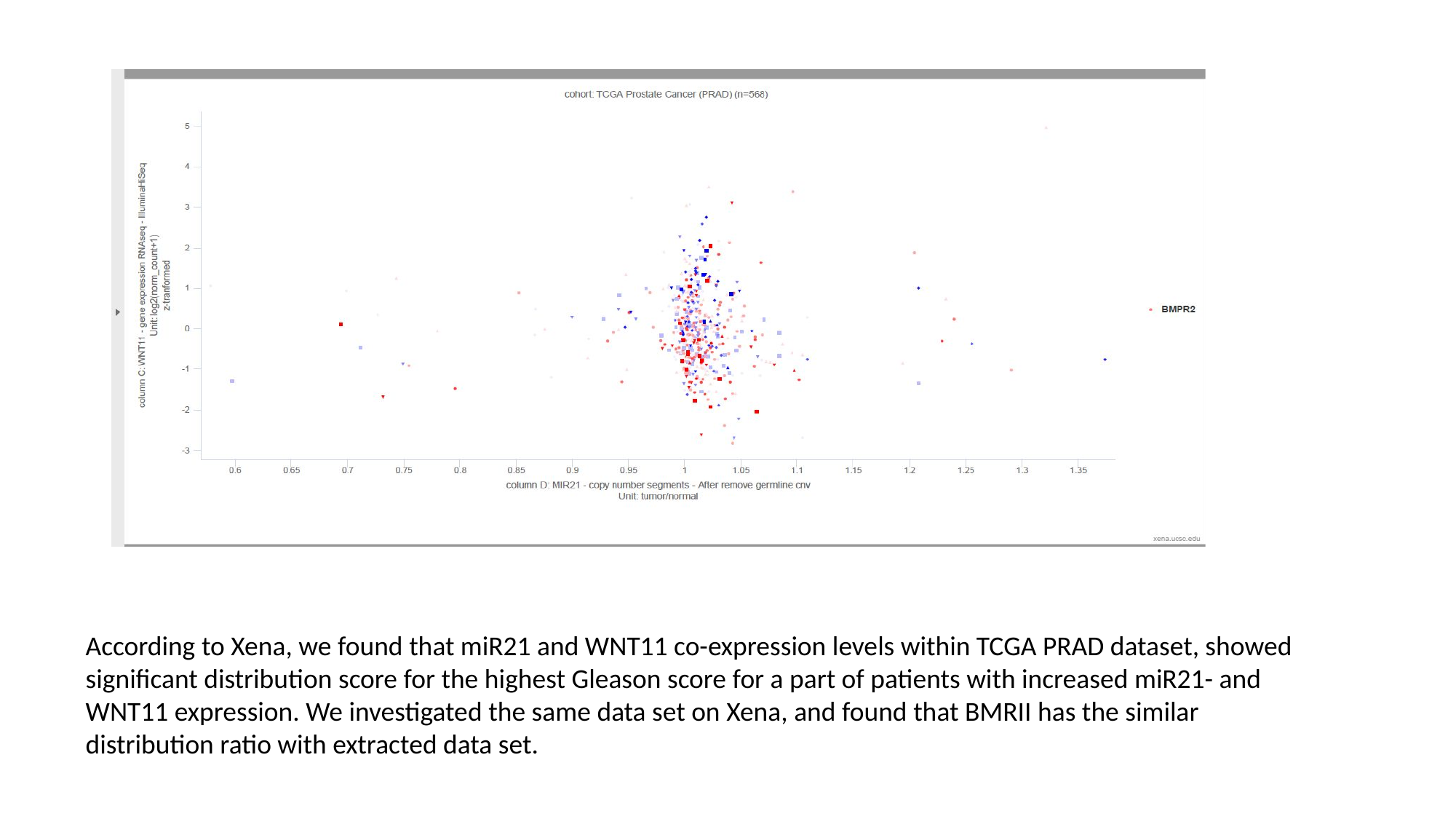

According to Xena, we found that miR21 and WNT11 co-expression levels within TCGA PRAD dataset, showed significant distribution score for the highest Gleason score for a part of patients with increased miR21- and WNT11 expression. We investigated the same data set on Xena, and found that BMRII has the similar distribution ratio with extracted data set.

## Slide 6
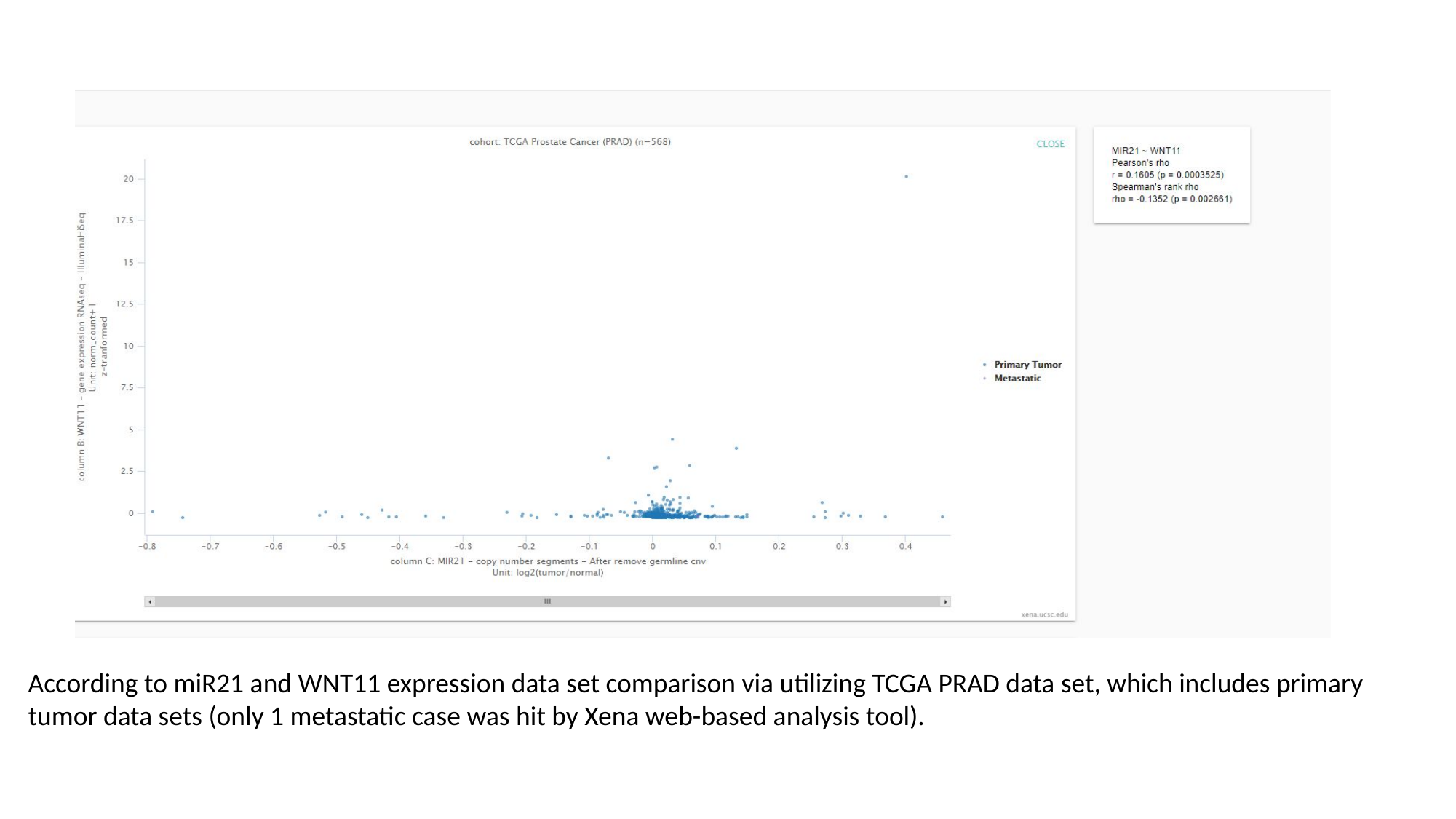

According to miR21 and WNT11 expression data set comparison via utilizing TCGA PRAD data set, which includes primary tumor data sets (only 1 metastatic case was hit by Xena web-based analysis tool).
